# Relationship between gait kinematics and walking energy expenditure during pregnancy in South African women

**DOI:** 10.1186/s13102-018-0100-x

**Published:** 2018-06-19

**Authors:** Zarko Krkeljas, Sarah Johanna Moss

**Affiliations:** 1 Physical Activity, Sport and Recreation Research Focus Area, North-West University, Private Bag x6001, Internal Box 481, Potchefstroom, 2520 South Africa; 2 Duke Kunshan University, 8 Duke Avenue, Kunshan, Jiangsu Province, 215316 China

**Keywords:** Gait, Pregnancy, Energy expenditure, Centre of gravity, Kinematics

## Abstract

**Background:**

Various musculoskeletal changes occurring during pregnancy may lead to the change in gait and contribute to the increase in walking energy expenditure. Previous research indicates that changes in gait mechanics may lead to the increase in mechanical work required during walking. However, there is little information to indicate if changes in gait mechanics during pregnancy have impact on active or total energy expenditure. Therefore, the primary aim of this study was to investigate the relationship between changes in gait kinematics and walking energy expenditure in pregnant women.

**Methods:**

Thirty-five women (mean age = 27.5 ± 6.1 years) volunteered for the study during various stages of pregnancy (1st trimester average = 12.1 ± 2.2 weeks; 2nd trimester = 22.3 ± 2.6 weeks; 3rd trimester = 31.4 ± 2.6 weeks). 3D motion analysis was used to assess changes in kinematic parameters during walking at self-selected pace. Resting metabolic rate, and walking energy expenditure expressed in terms of rate and cost of O_2_ were analysed with portable metabolic analyser.

**Results:**

Only medio-lateral deviation of centre of gravity (COG_ML_) increased 13.6% between the 1st and 2nd, and 39.3% between 2nd and 3rd trimester (*p* ≤ 0.001). However, self-selected walking speed depicted strong significant positive linear relationship with net O_2_ rate (*r* = 0.70; *p* ≤ 0.001), and was strongly associated with the vertical excursion of the COG (*r* = 0.75, *p* ≤ 0.001).

**Conclusions:**

Changes in gait mechanics during pregnancy may lead to an increase in walking energy expenditure. However, the consequent increase in walking energy cost may not be sufficient to offset the natural energy sparing mechanism.

## Background

Energy sparing during pregnancy is considered an inherent evolutionary biological mechanism [1]. There are numerous compensatory mechanisms that may be utilized to gain positive energy balance [2]. However, the energy required for foetal development is relatively small [3], and well-nourished mothers have adequate fat stores to provide for the additional energy needed for development. Although reducing the amount of walking or even reducing the walking speed are behavioural changes in daily activities that may result in reduction of total daily energy expenditure (TEE) [2], other changes in gait mechanics during pregnancy may lead to an increase in walking energy expenditure [4]. Yet, it is not clear if the relative energy changes as a result of alterations in gait mechanics have a significant impact on overall energy balance during pregnancy.

The change in trunk moment of inertia during pregnancy causes various compensations and adaptions in posture and gait mechanics that may result in the increase in walking energy expenditure [4]. As a significant portion of the metabolic cost of walking is attributed to the work required to move the body’s centre of mass (COM) [5, 6], any changes in gait kinematics affecting path of the COM would reflect on the energy expenditure [4]. It is well established that changes in gait kinematics are associated with changes in primary gait determinants [7, 8] which may lead to increase in metabolic cost of locomotion, yet there is a lack of studies investigating this relationship during pregnancy.

For example, self-selected walking speed decreases later in pregnancy [2, 9], which is associated with smaller trunk rotations [10], and consequent “flattening” of the COM which is associated with the decrease in walking metabolic cost. Additionally, step width increases during pregnancy which is associated with promotion of balance during walking [9, 11], but results in greater side-to-side movements of the COM leading to greater mechanical work and consequent increase in walking energy expenditure. Similarly, a decrease in stride length, an increase in double support time, and a decrease in step frequency have also been noted during pregnancy [9], and may be associated with changes in movement of the COM during walking. In addition, anterior weight distribution places an increased demand on the lumbar spine and the abdominal muscles, causing an anterior pelvic tilt and consequently lumbar lordosis commonly reported in pregnancy [12]. Postural adaptations will lead to anterior-posterior changes in COM [13].

On the other hand, changes in active and total energy expenditure during pregnancy may be interpreted through quality or quantity of movement. For example, since the net daily energy expenditure during pregnancy does not differ significantly from pre-pregnancy for the same activity [2, 3, 14–16], this indicates either a decrease in the pace of performing that activity [16], or an effective mechanical adaptation in the execution of a physical activity [2]. In addition, an increase in resting metabolic rate (RMR) during gestation and a simultaneous decrease in daily net oxygen consumption (VO_2_) may also be an indication of a strategy for a more economical movement [2, 16].

While various gait parameters have been investigated during pregnancy, only walking speed has been investigated relative to the energy expenditure [2]. Therefore, the primary aim of this study is to investigate the relationship between the gait kinematics and the metabolic cost of walking during pregnancy.

## Methods

This study was derived from a larger **H**abitual **A**ctivity **P**atterns during **P**regnanc**y** (HAPPY) study that investigated the changes in objectively determined physical activity patterns during pregnancy and their influence on various pregnancy outcomes. Thirty-five pregnant women at different stages of pregnancy, mean age 27.5 years (S.D. = 6.1), were recruited by advertisements in the local press, the consulting rooms of local gynaecologists, and a local health clinic in Potchefstroom, North West Province, South Africa. To participate in the study, women had to be healthy, between the ages of 18 and 40 years, without mental or physical disability, able to complete the test protocol, and not be considered a high-risk pregnancy according the guidelines of the American College of Sports Medicine (ACSM) [17]. Participants were allowed to return for additional measures at different stages of pregnancy. The women gave written consent for participation before data collection. A translator was available in the case of language barriers. The study was approved by the Human Research Ethics committee of North-West University (NWU-0044-10-A1).

Procedures for walking and resting energy expenditure, and gait analysis were previously described in Krkeljas and Moss [4], hence only a brief description of the methodology will be provided in the following section.

To measure RMR participants lay still for 5 min on their left side to ensure a resting state, after which Fitmate metabolic system (Cosmed Fitmate, Italy) was attached. RMR gas exchange was monitored for 16 min per Fitmate RMR protocol.

Walking energy expenditure was measured using the portable K4b^2^ (Cosmed, Italy) metabolic system, while participants walked at a self-selected pace along a 30-m-long oval track in the laboratory until steady state was reached. Steady state was considered by heart rate variation being no more than ±3 beats per minute (bpm), and less than 5% variation in respiratory quotient (RQ) [18], during which RQ of less than ≤0.99 had to be maintained [19]. The following parameters were recorded: walking volume of oxygen (VO_2_) (ml/kg/min), RQ, RMR (kcal/day), heart rate (bpm).

Full body 3D gait analysis was completed using eight Oqus 300+ cameras from Qualisys Motion Analysis System (Qualisys, Sweden) and collected at 220 Hz. Reflective markers were placed according to CAST/IK/HH (calibrated anatomical systems technique/Helen-Heyes/ inverse kinematics) gait model. During dynamic trials, participants were instructed to walk in a straight line at a self-selected pace along a 15 m laboratory walkway embedded with four AMTI BP400600 force plates (AMTI, Watertown, MA, USA). Motion and ground reaction force data were collected simultaneously for 5 s in the middle part of the runway. Only trials in which the participant’s foot landed entirely on a force plate for three consecutive steps (i.e. at full stride), were considered for inclusion in the data set. The subjects continued walking until three trials at full stride were completed. The participants were instructed to stop and rest as long as necessary, should they have felt tired at any stage of the examination of their gait. None did so.

### Data analysis

Gait kinematics analysed were: walking speed (time it takes to complete a single stride measured in m/s), step length (distance between reflective markers placed on Achilles tendon measured in meters) and step width (distance between left and right foot joint centres determined as the midpoint between lateral and medial malleoli measured in meters) normalized for leg length, double-support time (time from heel strike to push of the opposite foot measured in seconds), vertical and medio-lateral excursion of centre of gravity (COG)(m). The vertical force of 5% of body weight was used as a threshold for heel contact and toe-off.

During walking trials, the data were inspected for gaps in marker trajectories. The default gap-fill function was applied for gaps of no more than 10 frames using non-uniform rational basis spline (NURB) spline interpolation. No walking data trials analysed had gaps of more than 10 frames. Once the walking trials were limited to include only completed strides, the data were exported to Visual 3D-motion analysis software (C-Motion, MD, USA) for processing. The kinematic parameters were low-pass filtered with a bidirectional Butterworth filter with a 10 Hz cut-off frequency to remove noise from the differentiation process with zero-phase distortion [20].

Metabolic energy expenditure was reported as O_2_ consumption (O_2_ rate) (ml/kg/min), and to demonstrate the physiological work (O_2_ cost) for a given task by normalizing the O_2_ consumption for speed (ml/kg/m) [21]. In addition, to reduce the impact of changes in RMR O_2_ consumption was also analysed as net energy consumption by deducing the RMR from total energy expenditure. The net O_2_ cost may also be less sensitive to changes in walking speed [22]. This method in principle accounts for pregnancy-induced changes; however, nothing in the literature was found that addressed this normalization process in respect of gait in pregnancy.

### Statistical analysis

Data are presented as means ± standard deviation as specified. Shapiro-Wilks test was used to assess the data distribution. Levene’s test was used to determine whether there were any differences in variances between trimesters. A one-way ANOVA was used to assess differences between trimesters for the women’s physical characteristics, gait kinematics, and gait metabolic energy expenditure. LSD post hoc correction was set at α = 0.05 for all analyses. ANOVA was confirmed via a Kruskal–Wallis test for non-parametric data. If there were significant differences in variances between trimesters, Games–Howell post-hoc test was conducted. Pearson’s product correlations were computed to determine correlations between outcome variables. Therefore, the trimester of pregnancy is considered an independent variable, while kinematic and metabolic data are the dependent variables. All analyses were performed using SPSS v.21.0 (IBM Corp., Armonk, NY).

## Results

Participants’ descriptive parameters in respect of anthropometrics, gait kinematics, and energy expenditure per trimester are depicted in Table 1.

**Table 1:** Participants’ characteristics with respect to gait kinematics and walking energy expenditure per trimester

Measure	1st trim. Mean ± SD	2nd trim. Mean ± SD	3rd trim. Mean ± SD	Sig. (*p*)
Participants _(n)_^z^	14	20	10	
Age _(years)_	28.1 ± 5.5	27.1 ± 6.1	26.6 ± 6.6	0.83
Gestation _(wks)_	12.1 ± 2.2	22.3 ± 2.6	31.4 ± 2.6	–
Height _(cm)_	160.8 ± 5.9	160.2 ± 6.8	161.4 ± 7.2	0.89
Mass _(kg)_	62.7 ± 10.5	71.3 ± 16.6	78.8 ± 14.7	0.08
BMI _(kg/m_^2^_)_	24.3 ± 4.0	27.7 ± 6.2	29.9 ± 4.9	0.08
M_gain (kg)_	1.1 ± 3.1^a,c^	5.3 ± 2.8^b,c^	13.8 ± 7.9^a,b^	0.00^***^
S _(m/s)_	1.09 ± 0.07	1.10 ± 0.11	1.01 ± 0.19	0.16
Stride length^*^	0.69 ± 0.06	0.72 ± 0.06	0.70 ± 0.05	0.17
Step width^*^	0.06 ± 0.02	0.07 ± 0.02	0.07 ± 0.02	0.30
DS time _(s)_	0.12 ± 0.03	0.11 ± 0.03	0.13 ± 0.06	0.35
COG_V (cm)_	3.37 ± 0.56	3.55 ± 0.73	3.22 ± 0.726	0.53
COG_ML (cm)_	2.06 ± 0.42^a^	2.34 ± 0.89^b^	3.26 ± 0.57^a,b^	0.001^**^
REE (_kcal/day)_	1405.7 ± 183.7	1488.1 ± 190.0	1578.0 ± 216.1	0.12
REE _(kcal/kg/day)_	22.7 ± 2.6	21.4 ± 2.5	20.9 ± 2.2	0.40
Gross O_2 (ml/kg/min)_	10.93 ± 2.46	9.66 ± 1.45	10.39 ± 2.01	0.26
Gross O_2 (ml/kg/m)_	0.17 ± 0.04	0.15 ± 0.02	0.17 ± 0.03	0.16
Net O_2 (ml/kg/min)_	9.15 ± 3.26	8.04 ± 2.72	8.51 ± 3.69	0.60
Net O_2 (ml/kg/m)_	0.12 ± 0.04	0.10 ± 0.02	0.12 ± 0.02	0.16
RQ	0.90 ± 0.11^b^	0.89 ± 0.06^a^	0.96 ± 0.02^a,b^	0.04^*^

^a,b,c^ denotes significance between respective trimesters; M_gain_ = mass gain from pre-pregnancy (i.e. total mass gain); S = walking speed; DS = double support; COGv = vertical excursion of the centre of gravity; COG_ML_ = medio-lateral centre of gravity displacement;^*^ = normalized for leg length; O_2_ = walking volume of oxygen; RQ = respiratory quotient; Net O_2_ = energy expenditure only necessary for walking (TEE_gait_ - REE); trim. = trimester; ^z^ Several participants were measured in multiple stages

Coefficient of variation in first trimester for all metabolic and kinematic variables ranged from 6.4% for walking speed to 35.2% for net walking energy expenditure. Weight gain per trimester was within the range recommended by the Institute of Medicine (6.7–11.2 kg) [23]. Based on self-reported pre-pregnancy weight, participants were on average borderline overweight with a mean of 25.1 ± 5.5 kg/m^2^ [23].

Differences between the trimesters in gross and net walking energy expenditure were not significant. Similarly, while absolute REE was greater in each consequent trimester, the differences were not statistically significant. When normalized for the mass gain REE was decreasing, although these differences were also not statistically significant. The mean respiratory quotient (RQ) remained below 1.0 (mean = 0.91 ± 0.07) an indication of aerobic metabolism dominant throughout pregnancy [21, 24]. However, the RQ was significantly higher in the 3rd trimester (*p* ≤ 0.05) and was close to 1.0 (0.96 ± 0.02), which signifies a potential change in metabolic process.

Relative to gait kinematics, only COG_ML_ significantly increased between trimesters (*p* ≤ 0.001), while walking speed, step length, and step width remained unchanged (Table 1). Changes in gait kinematics, step width and COG_ML_ were associated with mass gain rather than the absolute mass (*r* = 0.38, *p* ≤ 0.01 and *r* = 0.50, *p* ≤ 0.001 respectively) (Table 2), whereas changes in walking speed were inversely related to the mass (*r* = − 0.43, *p* ≤ 0.001). However, relative to the net energy rate and cost, only self-selected walking speed (*r* = 0.70, *r* = 0.53, *p* ≤ 0.001, respectively) and COGv (*r* = 0.45, *p* ≤ 0.01 and *r* = 0.30, *p* ≤ 0.05) showed significant association.

**Table 2:** Pearson correlations between body weight, gait kinematics and walking energy expenditure

	COG_V (m)_	COG_ML (m)_	GR O_2 (ml/kg/min)_	NR O_2_ (ml/kg/min)	NC O_2_ (ml/kg/m)	Mass _(kg)_	M_gain_ (kg)
Mass _(kg)_	−0.18	0.24	−0.15	−0.19	− 0.11	–	–
M_gain (kg)_	−0.01	0.50^***^	−0.04	− 0.08	−0.02	0.43^**^	–
S (m/s)	0.75^***^	−0.18	0.30^*^	0.70^***^	0.53^***^	−0.43^**^	−0.27
Stride length^a^	0.32^*^	−0.39^**^	−0.18	0.09	−0.01	− 0.01	−0.29
Step width^a^	0.09	−0.02	0.20	0.19	0.22	0.05	0.38^**^
DS time _(s)_	−0.17	0.00	0.23	−0.23	−0.18	0.15	0.34^*^
COG_V (m)_	–	–	0.11	0.45^**^	0.30^*^	−0.18	− 0.01
COG_ML (m)_	−0.03	–	− 0.10	−0.12	− 0.09	0.24	0.50^***^
REE _(kcal/day/kg)_	–	–	0.04	0.03	−0.04	−0.85^***^	− 0.31^*^

^*^p ≤ 0.05; ^**^ ≤ 0.01; ^***^ ≤ 0.001; M_gain_ = mass relative to pre-pregnancy mass; S = walking speed; DS = double support; COGv = vertical excursion of the centre of gravity; COG_ML_ = medio-lateral centre of gravity displacement; ^a^normalized for leg length; GR O_2_ = gross O_2_ rate; NR O_2_ = net O_2_ rate; NC O_2_ = net O_2_ cost; REE = resting energy expenditure

**Fig. 1:**
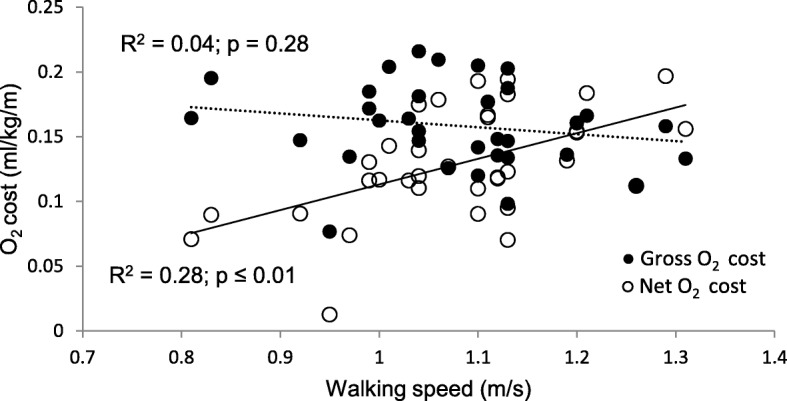
Gross and net energy cost relative to walking speed during pregnancy

**Fig. 2:**
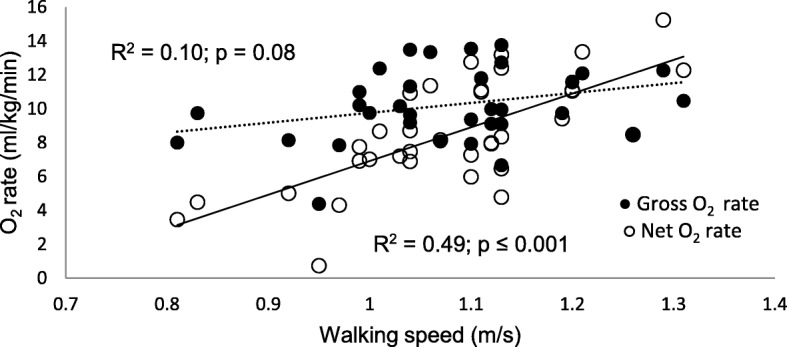
Changes in gross and net energy rate relative to changes in walking speed during pregnancy

Net walking energy cost and rate are significantly associated with walking speed (Figs. 1 and 2, respectively), whereas gross energy expenditure shows weak and non-significant relationship.

## Discussion

The principal findings of this study are two-fold: firstly, self-selected walking speed has strong significant relationship with net walking energy expenditure during pregnancy; and secondly, the relative mass gain, rather than the absolute mass is a primary factor associated with changes in gait mechanics which may lead to increase in walking energy expenditure.

In this study, although the differences between trimesters in gait kinematics and walking energy expenditure were not statistically significant, there were significant associations between gait kinematics and walking energy expenditure. Similarly to previous studies, self-selected walking speed during third trimester was lower than the first or second trimesters [2, 9], although in this study this decrease was not statistically significant. Changes between trimesters in gross and net walking energy expenditures were also not significantly different. However, self-selected walking speed showed strong significant relationship with net walking energy rate (ml/kg/min) and walking economy (ml/kg/m) (Figs. 1 and 2, respectively), while there was a lack of association with the gross energy expenditure. Since gross energy expenditure contains REE, the variability in REE which is associated with physiological changes due to foetal development, would not be related to the energy expenditure required for walking. Increase in resting energy expenditure (kcal/day) (Table 1) is associated with the increase in mass (Table 2), although the lack of statistical differences may be attributed to the large variability in mass gain between the participants, or the differences in self-reported pre-pregnancy weight.

The relationship of speed of walking and net energy expenditure is largely determined by the COG_v_ (*r* = 0.70, *p* ≤ 0.001; *r* = 0.45, *p* ≤ 0.01, respectively) (Table 2). Given that the motion of the COG may be regarded as the summation of all forces that act on the body, the significant portion of the total metabolic cost during walking should be attributed to the work required to move the COG [5, 6], especially as the weight of the body increases as in pregnancy. This effect has been demonstrated in our previous article [4]. This relationship indicates that the ability to increase walking efficiency is related to the principle of conservation of mechanical energy during walking that is maximized at certain speeds [4, 5, 25], which participants in this study did not reach. The average self-selected walking speed of 1.08 ± 0.11 m/s did not significantly change during pregnancy and falls within previously reported range from 0.83 m/s [10] to 1.5 m/s [2].

While the changes in walking speed were associated with the absolute mass (*r* = − 0.43, *p* ≤ 0.01), gait parameters associated with the greater stability during walking, step width and the time spent in double-support stage, were associated with the relative mass gain (*r* = 0.38, *p* ≤ 0.01 and *r* = 0.34, *p* ≤ 0.05, respectively). Due to weight distribution during pregnancy, the trunk moment of inertia increases leading to need for greater stability [20]. More stability during walking may be obtained by increasing double-support time, increase the step width, or both, in order to create a larger base of support. In addition, lower walking speeds results in an increased double support time, which gives pregnant women more time to react and control additional balance demands during walking [9, 20, 26].

However, these gait changes may result in mechanically inefficient gait which may lead to increase in total energy expenditure [4]. Walking with the bigger base of support results in large side-to-side excursions of the centre of gravity (COG) [26], which may increase the energy demand as discussed earlier. The results in this study show 13.6% increase of medio-lateral excursions of centre of gravity (COG_ML_) between the first and second trimester, and 39.3% between second and third trimester (*p* ≤ 0.001). These changes were significantly related to relative mass gain (*r* = 0.50, *p* ≤ 0.001), rather than the absolute mass. In late pregnancy, due to large mass gain, width of the pelvic girdle also increases in order to accommodate the growing foetus, which also leads to the increase in the width of the base of support [11] and consequently the increase in the step width during pregnancy [27].

While changes in gait mechanics may have a significant impact on walking energy expenditure, the metabolic cost of walking may not be sufficient to alter the overall net energy balance. The increase in absolute REE between the trimesters (although not statistically significant) was largely associated with the mass (*r* = 0.86, *p* ≤ 0.001), however, once normalized for the mass REE decreased between subsequent trimesters and showed strong negative correlation with the mass (*r* = − 0.85, *p* ≤ 0.001), which is suggestive of energy conservation process during pregnancy associated with the changes in metabolism [2]. However, the difference in REE between the 1st and 3rd trimester was 1.8 kcal/kg, indicating that energy sparing process in a woman with approximate weight of 65 kg (average pre-pregnancy weight in this study = 64.4 ± 14.7 kg), would conserve 117 kcal/day – only a 6.5 to 5.9% increase from 1800 to 2000 kcal/day recommended daily caloric intake for healthy women of the same group and activity level as reported in this study. Considering the relationship of walking speed and net energy expenditure in this study, and the decrease from 1st to 3rd trimesters in walking speed, the difference in energy expenditure conservation by means of walking would equal to 0.5 kcal/min for the same individual. Therefore, for conservation of energy from changes in gait to have a meaningful impact on overall energy expenditure during pregnancy, women would have to walk continually for several hours.

The small impact changes in gait mechanics have on total energy expenditure, allows for gait mechanics to be altered for reasons such as balance or comfort, which may lead to mechanically inefficient gait [4], but without the significant impact on overall energy expenditure, which helps maintain overall net positive energy balance during pregnancy. Because pregnancy is characterized by the bearing of an extra and “valuable” load, and as such walking efficiency has to be combined with safety. While the additional burden of the growing fetus may increase the demand of mechanical energy, women tend to adopt a strategy that helps them maintain the rate of energy expenditure at a level that can be sustained for a relatively long time. This is also a strategy adopted by individuals who walk with a pathological condition [28]. Considering that the pre-pregnancy physical and physiological characteristics differ among the women studied, this is also the most likely source of large inter-subject variability in gait parameters during pregnancy reported across all similar studies.

The results of this study have to be considered in regard to the limitations presented during data collection. Firstly, not all the pre-pregnancy weight was obtained from participants’ records and was therefore self-reported, which is known to be under-estimated at the times. Secondly, large withdrawal rates prevented longitudinal tracking, which would allow identification of the most common changes occurring during pregnancy in the parameters investigated.

## Conclusion

The changes in gait mechanics during pregnancy may occur as a result of various adaptations and needs of the mother. It is likely that those changes will result in change in energy expenditure during walking. However, considering the inherent energy conservation process occurring during pregnancy, the changes in energy expenditure due to gait are not sufficient to significantly alter the overall positive energy balance.

## Abbreviations

BOS: Base of support
COG: Centre of gravity
COG_ML_: Medio-lateral excursions of the centre of gravity
COM: Centre of mass
GR: Gross rate
NC: Net cost
NR: Net rate
O_2_: Oxygen (molecular formula)
PA: Physical activity
REE: Resting energy expenditure
RMR: Resting metabolic rate
RQ: Respiratory quotient
TEE: Total energy expenditure
VO_2_: Volume of oxygen consumption
